# TRPM8-driven thermogenesis by menthol: mechanisms of cold injury prevention

**DOI:** 10.1007/s13105-025-01120-8

**Published:** 2025-09-13

**Authors:** Yujie Li, Yuanyuan Song, Xin Yang, Haiwei Zhu, Hao Yu, Yuan Kong

**Affiliations:** 1 Department of Endocrinology, General Hospital of Northern Theater Command, No. 83 Wenhua Road, Shenyang, 110000 Liaoning Province China; 2 Department of Endocrinology, The Second Affiliated Hospital of Baotou Medical College, Baotou, 014030 China; 3 Department of Special Service, The 960th Hospital of the PLA Joint Logistics Support Force, Jinan, 250000 China

**Keywords:** Menthol, TRPM8, Brown adipose tissue, Thermogenesis, Cold injury, Network pharmacology

## Abstract

Cold injury presents a significant health challenge, causing tissue damage due to prolonged exposure to low temperatures. This study examines menthol’s protective effects against cold injury, focusing on its activation of transient receptor potential cation channel subfamily M member 8 (TRPM8), a “cold-sensing” receptor, to stimulate thermogenesis in brown adipose tissue (BAT). Male C57BL/6J mice were treated with menthol for 21 days and exposed to -20 °C. Core body temperature, activity levels, and cold injury severity were measured. Network pharmacology methods identified TRPM8 as a potential target, confirmed through molecular docking and pathway analysis. Further experiments inhibited TRPM8 to evaluate its role in menthol-induced thermogenesis and cold tolerance. Menthol significantly raised core body temperature, improved cold tolerance, and reduced cold injury severity in treated mice. Network pharmacology analysis highlighted TRPM8 as a key regulator of BAT thermogenesis through the PKA/UCP1 pathway. TRPM8 inhibition diminished menthol’s effect, underscoring its essential role in menthol-mediated thermogenesis. This study demonstrates that menthol activates TRPM8 in BAT, enhancing thermogenesis to prevent cold injury. These findings suggest menthol as a promising natural agent for cold injury prevention, with TRPM8 as a potential therapeutic target.

**Supplementary Information:**

The online version contains supplementary material available at 10.1007/s13105-025-01120-8.

## Graphical Abstract

**Figure:**
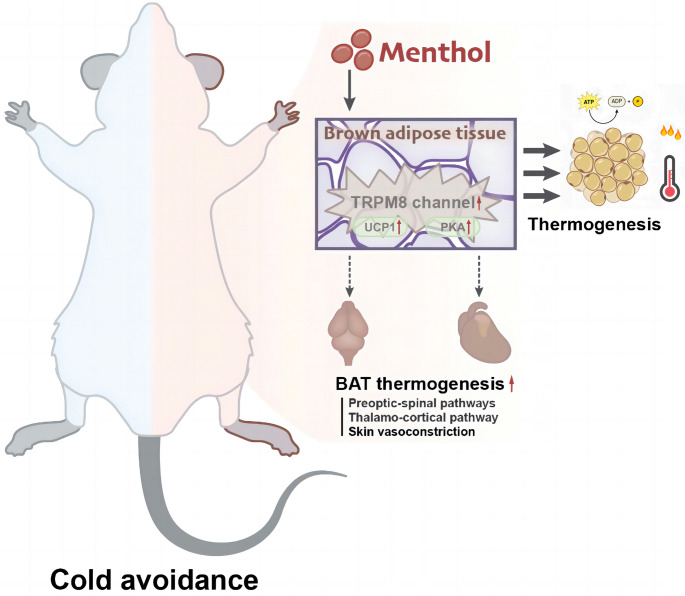
Mechanism of action illustrating how menthol induces BAT thermogenesis to prevent cold injury by activating the “cold channel” TRPM8

## Introduction

Cold injury is a condition caused by exposure to low temperatures, leading to tissue freezing, electrolyte imbalances, pH fluctuations, blood clot formation, vasoconstriction, microvascular damage, and release of inflammatory cytokines, ultimately resulting in cell death and tissue damage. It is characterized by a high incidence rate and a complex treatment process. Upon rewarming, reperfusion injury can further exacerbate tissue damage [1, 2]. The severity of cold injury primarily depends on ambient temperature and duration of exposure, with higher prevalence in cold climates and high-altitude areas [3]. Clinical management of cold injuries involves rapid diagnosis, environmental transfer, prompt rewarming, anti-inflammatory therapy, sterile wound care, thrombolytic treatment, and early orthopedic and plastic surgical consultations [4]. Treatment mechanisms focus on transferring the patient to a warm environment and swiftly rewarming to halt the cold-induced damage and facilitate adequate reperfusion. Anti-inflammatory therapy aims to mitigate the detrimental effects of prostaglandins in cold-injured tissues and reduce inflammation. Vasodilators and thrombolytics are used to enhance perfusion in damaged areas and restore blood flow, while sympathetic nerve blockade aids in restoring skin temperature and vasodilation in the affected extremities. Amputation may be necessary to remove necrotic tissue and protect healthy limbs from infection [5].

Menthol exhibits a broad range of effects and can be utilized for alleviating itching, managing chronic obstructive pulmonary disease (COPD), preventing chilblains, and treating local inflammation, pain, rhinitis, and cough [6, 7]. As a partial agonist of the specific cold receptor protein TRPM8, it enhances the body’s perception of cold [8]. By increasing the activity of myosin light chain phosphatase (MLCP) to inhibit the activation of myosin light chain kinase (MLCK) mediated by calcium-binding proteins, it suppresses the RhoA/ROCK pathway, blocks the activation of L-type voltage-gated calcium channels, and increases NO release alongside TRPM8 activation, resulting in vascular smooth muscle (VSM) relaxation [6]. In cases of nerve damage or chemical stimulation, menthol activates TRPM8 to alleviate mechanical and thermal pain sensations [9]. Menthol also increases the activity of antioxidant enzymes such as superoxide dismutase (SOD), glutathione reductase (GR), and glutathione peroxidase (GPx), as well as the level of glutathione (GSH), while decreasing levels of inflammatory cytokines, including TNF-α, IL-6, and IL-1β [10].

Brown adipose tissue (BAT) plays a vital role in thermoregulation and thermogenesis by generating heat through its unique energy metabolism process, thus effectively combating cold-induced damage [11]. Exposure to cold or β3-adrenergic receptor stimulation can induce the production of 12-lipoxygenase (12-LOX) in BAT, leading to an increase in 12-hydroxyeicosatetraenoic acid (12-HEPE). This 12-HEPE activates the PI3K/mTOR/Akt/Glut pathway, enhancing the expression of de novo lipogenesis genes and glucose transporter-1, which provides fatty acids for uncoupling protein 1 (UCP1) activation [12]. Additionally, BAT can produce MaR2, which alleviates inflammation by reducing TNF-α levels [13]. Activation of the PKA pathway in BAT regulates the activation of PGC-1α and thermogenic genes, and UCP1 facilitates proton transfer to the mitochondrial matrix, thus promoting thermogenesis through uncoupled oxidative phosphorylation [14, 15]. Cold exposure can also enhance TRPM8 expression in BAT, promoting thermogenesis [16].

Activation of TRPM8 sensitizes the cold-sensing receptors, mimicking environmental cold exposure in the body [9], triggering the release of norepinephrine by adrenergic neurons that control BAT, and inducing UCP1 thermogenesis [17]. The involvement of TRPM8 in painful hypersensitivity reactions indicates that using direct antagonists and excessive agonists can lead to channel deactivation, alleviating the body’s perception of pain [18]. Activation of TRPM8 can decrease the levels of pro-inflammatory cytokines TNF-α, IL-1β, IL-6, and the chemokine MCP-1, thus preventing inflammatory infiltration [19], as well as increasing the release of NO to relax VSM [6]. Recent studies have found that menthol, by activating the TRPM8 channel, can enhance BAT’s thermogenesis capacity, potentially offering preventive effects against cold injuries [17].

Therefore, this study employs network pharmacology methods to investigate the mechanisms by which menthol activates the TRPM8 channel to promote BAT thermogenesis, evaluating its potential to prevent cold injury. This research aims to provide novel insights and experimental evidence for the prevention and treatment of cold injuries.

## Materials and methods

### Experimental animals

All experiments involving mice were approved by the Animal Ethics Committee of the General Hospital of Northern Theater Command (No. CMUXN2022801) and strictly followed the guidelines for the Care and Use of Laboratory Animals [20]. Forty 18–22 g male C57BL/6J mice were purchased from Hunan Slaijinda (Hunan, China). The mice were acclimatized for one week in an environment with a temperature maintained at 30 °C and a light/dark cycle of 12 h/12 hours. The temperature of 30 °C closely approximates the thermoneutral zone for mice in laboratory conditions, reducing energy expenditure on temperature regulation and allocating more energy to maintaining basic physiological needs. This setup helps to simulate conditions similar to room temperature, providing a stable baseline for studying the impact of menthol on BAT thermogenic capacity. The mice were provided with a set amount of food intake each day (3.5 g per mouse), allowing for ad libitum access to food and water. All experiments were conducted during the light phase to minimize disruption to the animals’ physiological rhythms.

Additionally, C57BL/6J background TRPM8 knockout (TRPM8 KO) mice were obtained from the Jackson Laboratory (JAX, stock no. 008198, USA) and backcrossed several generations to the C57BL/6 N background to establish wild-type mice (WT, *n* = 24) and TRPM8 KO mice (*n* = 16). The mice were individually housed at room temperature (30 °C) in shoebox-style cages lined with corn cob bedding. TRPM8 KO mice were born and raised at 30 °C, while the C57BL/6J mice purchased from JAX required at least one week of acclimatization to room temperature before being used for experiments [17]. The mice had ad libitum access to water and standard rodent chow and were used for subsequent studies at 8–12 weeks of age.

### Animal treatment

In Experiment 1, C57BL/6J mice were randomly divided into five groups, with 8 mice in each group. The experimental groups were treated with different concentrations of menthol (1%, 5%, 10%) and exposed to cold stimulation at -20 °C. The menthol solution (#M0350000, Sigma, USA) was prepared using 100% ethanol as a solvent, administered orally by gavage without fasting the animals before dosing. Throughout the experiment, the mice were treated at the same time each day (from 13:00 to 14:00) for a duration of 21 days. Cold exposure treatment started on the 15th day of dosing, where all groups of mice, except the control group, were exposed to a -20 °C environment for 15 min daily for 7 consecutive days. On the 21st day, a 2-hour long cold exposure test was conducted [20, 21].

Cold exposure treatment: Starting from the 15th day of dosing, all mice, except for the control group, were transferred to individual cages (30 × 30 × 30 cm) maintained at a temperature of -20 °C, humidity of 60%, and no airflow for 15 min daily over 7 days. On the 21st day, the animals were placed at -20 °C for 2 h [20].

Animal Experiment 2: WT mice and TRPM8 KO mice were divided into 6 groups, including the WT-control group, TRPM8 KO group, WT-cold exposure group, WT-high-dose menthol (10%)-cold exposure group, TRPM8 KO-cold exposure group, and TRPM8 KO-high-dose menthol (10%)-cold exposure group, with 8 mice in each group. The WT-control group, WT-cold exposure group, and TRPM8 KO-cold exposure group were administered an equal amount of control solution, while the WT-high-dose menthol (10%) group and TRPM8 KO-high-dose menthol (10%) group received a high dose of menthol (10%) treatment. The administration duration and method were the same as in Animal Experiment 1. The treatment lasted for 21 days, administered once daily [20, 21].

Cold exposure treatment: Starting on the 15th day of administration, except for the control group, all groups of mice were transferred to -20 °C for 15 min daily for 7 days. On the 21st day, the animals were exposed to -20 °C for 2 h [20].

### Temperature measurement

Following cold exposure treatment, an infrared digital thermographic camera (FLIR E5, FLIR, USA) and FLIR Quick Report software were used to conduct thermal imaging on the abdominal region of mice to capture and analyze surface temperature. Core body temperature was measured rectally post-anesthesia using a digital thermometer (# BAT-12, Worldunion Tech, China). Upon completion of temperature measurement, the mice were euthanized, and subsequent dissection was promptly performed to collect BAT, particularly from the neck and back regions (the back BAT is located bilaterally along the spine, especially in the thoracic and lumbar areas. The neck BAT is located in the interscapular region). The collected BAT was immediately stored at -80 °C for further biomarker analysis [20].

### Measurement of adenosine triphosphate (ATP) content

The ATP levels in BAT were measured using the “ATP content detection kit” (Beyotime Biotechnology Company, #S0026) following the recommended experimental protocol. The luminescence was detected using a fluorescence microplate reader (Synergy HT, LabX). The protein concentration was determined using the BCA protein concentration detection kit (Beyotime, P0012S). The ATP levels were expressed as nmol/mg of protein [20, 22].

### Hematoxylin and eosin (H&E) staining

BAT tissues were collected and fixed in 4% paraformaldehyde for 24–48 h, washed with phosphate-buffered saline (PBS), treated with 70% ethanol, and embedded in paraffin the following day. Tissue blocks were sliced into 5 μm sections, followed by staining with H&E. The sections were stained in hematoxylin solution (Sigma, #H3136) for several minutes, differentiated in acidic water and ammonia water for several seconds. After rinsing with tap water and soaking in distilled water for a period, the sections were dehydrated in 70% and 90% alcohol for 10 min each and then stained in eosin staining solution (Sigma, #318906) for 2–3 min. Stained sections were dehydrated with absolute alcohol, permeated with xylene, mounted with mounting medium, and sealed with a coverslip. Finally, the tissue sections were observed under a microscope (Eclipse E100, Nikon, Japan). Images were obtained from three random areas of each Sects. [20, 23, 24].

### Network pharmacology and bioinformatics analysis for identification of potential targets for menthol in preventing cold injury

Target Gene Screening for Menthol: Two forms of menthol, L-Menthol (PubChem CID: 16666) and DL-Menthol (PubChem CID: 1254), were retrieved from the PubChem database (https://pubchem.ncbi.nlm.nih.gov/) along with their Canonical SMILE (Simplified Molecular Input Line Entry System) structures. The prediction of menthol targets was carried out using the SwissTargetPrediction database (http://www.swisstargetprediction.ch/).

Key Target Selection: The GeneCards database (https://www.genecards.org/) was utilized to search and obtain a collection of genes related to “Cold injury” and “Cold stress.” The top 100 genes in terms of Relevance score from each collection were intersected with the menthol target genes to identify the key targets of menthol in preventing cold injury [25, 26].

### Construction of target gene interaction network

The protein-protein interaction network of target genes was obtained using the String database (https://string-db.org, version 12.0) with species limited to “Homo sapiens”. A regulatory relationship network was constructed and further imported into Cytoscape (v3.8.2) software for adjustment and visualization [25]. Parameter settings were kept at default values unless otherwise specified.

### Functional enrichment analysis of target genes

The target genes were subjected to Gene Ontology (GO) enrichment analysis using the “ClusterProfiler” package in R language (version 4.3.1), covering the analysis of biological process (BP), molecular function (MF), and cellular component (CC), with a significance threshold set at *P* < 0.05. Additionally, the Kyoto encyclopedia of Genes and Genomes (KEGG) pathway enrichment analysis was performed on the target genes using online tools as described in a study [25].

### Molecular simulation docking

The crystal structure of TRPM8 (PDB ID: 7WRA) was selected and downloaded from the Protein Data Bank. The three-dimensional structures of L-Menthol and DL-Menthol were obtained from the TCMSP database (https://old.tcmsp-e.com/tcmsp.php) and subjected to energy minimization using the MM2 algorithm. Subsequently, the receptor protein TRPM8 underwent dehydration and removal of organic molecules using PyMOL software. The receptor protein molecules were preprocessed using AutoDockTools 1.5.6, which included processes such as hydrogen addition, charge calculation, and conversion of compounds and receptor proteins to “pdbqt” files. Appropriate box center and grid parameters were set for the docking box. Finally, Vina 1.1.2 was utilized to evaluate molecular docking and calculate the docking energy values [27].

### Western blot analysis

The cells were lysed in RIPA lysis buffer (#P0013B, Beyotime Biotechnology, Shanghai, China). Protein concentration was quantified using the BCA (Bicinchoninic Acid) assay kit (A53226, Thermo Fisher Scientific, Rockford, IL, USA). Proteins separated by polyacrylamide gel electrophoresis were then transferred to a PVDF (Polyvinylidene Fluoride) membrane (IPVH85R, Millipore, Darmstadt, Germany) using the wet transfer method. The membrane was blocked at room temperature with 5% BSA (Bovine Serum Albumin) for 1 h and then incubated overnight at 4 °C with the primary antibody (specific antibody information can be found in Table S1). Subsequently, the membrane was washed and incubated with an HRP (Horseradish Peroxidase)-conjugated secondary IgG antibody for 2 h. The membrane was washed with TBST for 5 min thrice, followed by chemiluminescent detection. Protein quantification analysis was performed using ImageJ 1.48u software (v1.48, National Institutes of Health, USA) by calculating the ratio of the grayscale values of each protein to the internal control α-Tubulin [28]. Each experiment was conducted in triplicate.

### Statistical analysis

Statistical analysis and graphical representation were performed using GraphPad Prism (version 5). All data were presented as mean ± standard deviation (SD), and the analysis was carried out using analysis of variance (ANOVA) followed by Tukey’s post hoc test [20]. Box plot analysis was utilized to identify outliers, whereby any data point exceeding 1.5 times the interquartile range (IQR) was considered an outlier. Outliers deemed as experimental errors were excluded from the analysis. Multiple imputation was employed to address missing data, ensuring the completeness and accuracy of the dataset for analysis.

## Results

### Menthol enhances cold tolerance in mice exposed to cold environment

**Fig. 1:**
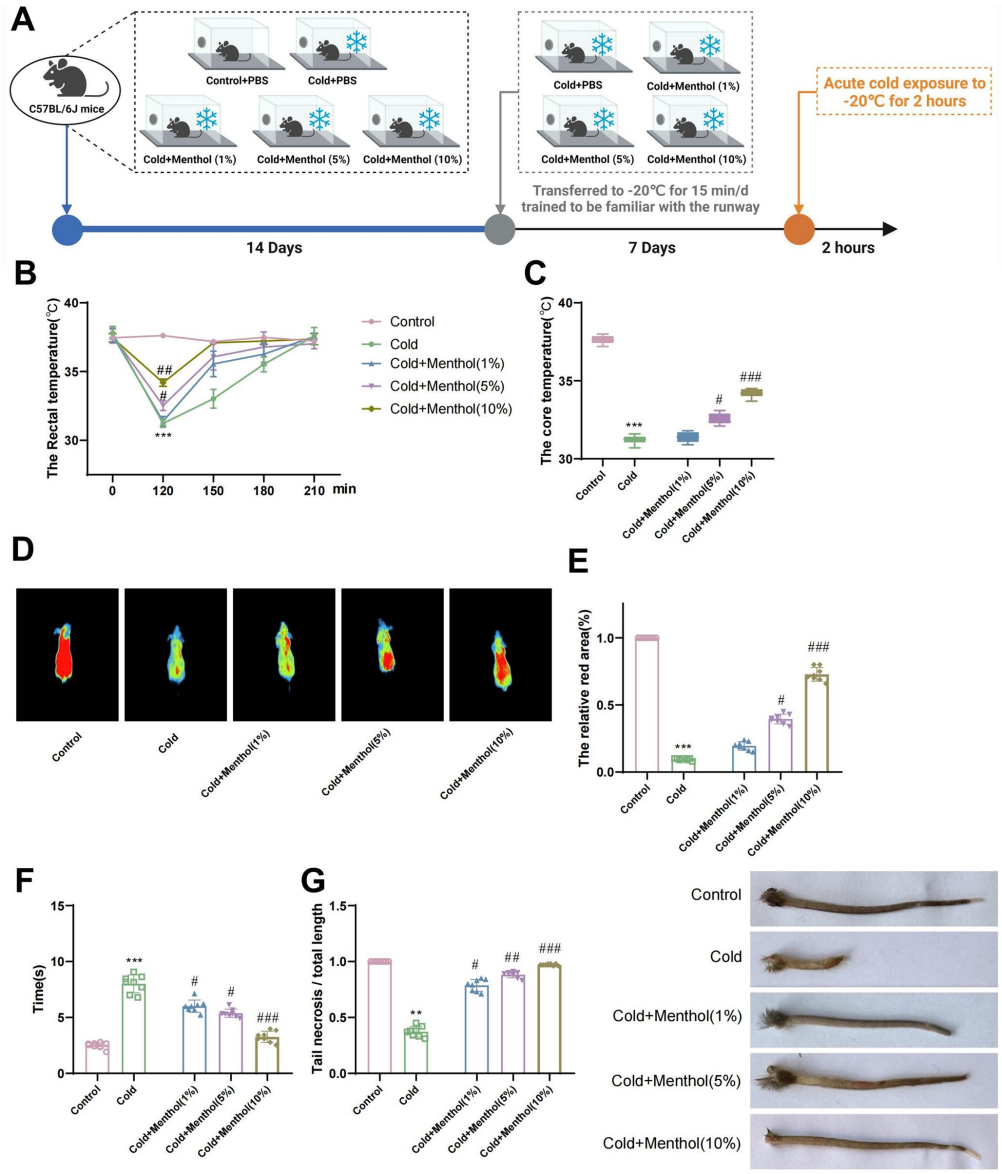
The effect of menthol on cold tolerance in exposed mice

To investigate the role of menthol in cold injury, we fed male C57BL/6J mice a normal diet and subjected them to daily oral gavage treatment with ethanol or menthol (at three doses: low (1%), medium (5%), and high (10%)) for 21 consecutive days, followed by exposure to -20 °C for 2 h (Fig. 1A). At 120 min, the mice from each group were retrieved and placed at room temperature. Subsequently, as the mice transitioned from the cold environment to room temperature, their blood vessels rapidly dilated, enhancing blood circulation and causing a temporary increase in body temperature before returning to normal levels. Therefore, the quicker the body temperature recovers, the greater the cold tolerance is.

At room temperature, as shown in Fig. 1B-C, compared to the control group, the rectal and core temperatures of mice in the cold-exposed group significantly decreased. Mice treated with the low dose (1%) of menthol showed no significant statistical differences in rectal and core temperatures compared to the cold-exposed group, while mice treated with medium (5%) and high (10%) doses of menthol exhibited significant increases in rectal and core temperatures. These findings indicate that menthol can maintain both the core and surface temperatures of mice in a cold environment, mitigate the decrease in core temperature, and facilitate temperature recovery following cold exposure.

Two hours after exposure, all mice reached their lowest core body temperature. Thermal imaging (Fig. 1D-E) indicated a significant decrease in surface body temperature in the cold-exposed group compared to the control group. Furthermore, mice treated with a low dose (1%) of menthol showed no significant difference in surface body temperature compared to the cold-exposed group, while mice treated with medium (5%) and high doses (10%) of menthol exhibited improved cold tolerance in terms of surface body temperature compared to other mice.

Additionally, varying degrees of tremors were observed in the bodies of mice from all groups in the cold environment, especially in the cold-exposed group. However, after a period of time at room temperature, they were able to regain normal activity. Mice with higher degrees of cold injury exhibited tail necrosis, with the necrotic part changing from pink to deep purple after cold exposure. Subsequently, the mice’s activity levels and degree of cold injury were further assessed. Following 2 h at -20 °C, mice in the cold-exposed group were unable to run quickly and only managed to move forward slowly. In contrast, mice in the menthol-treated group showed higher activity levels.

As shown in Fig. 1F, mice in the cold-exposed group took significantly longer to cross the runway compared with the control group. In contrast, mice treated with low (1%), medium (5%), and high (10%) doses of menthol spent less time crossing the runway than the cold-exposed group, with the time decreasing as the dose increased, indicating enhanced activity in menthol-treated mice. Subsequently, by measuring the original length of the tail and the length of the non-necrotic portion, the relative tail length of the mice was calculated, as shown in Fig. 1G. Mice in the cold-exposed group had shorter relative tail lengths compared to the control group. However, mice treated with low (1%), medium (5%), and high doses (10%) of menthol showed longer relative tail lengths than the cold-exposed group. These findings suggest that menthol enhances post-cold exposure activity in mice and reduces the degree of cold injury.

### Menthol target genes associated with multiple BP and signaling pathways

**Fig. 2:**
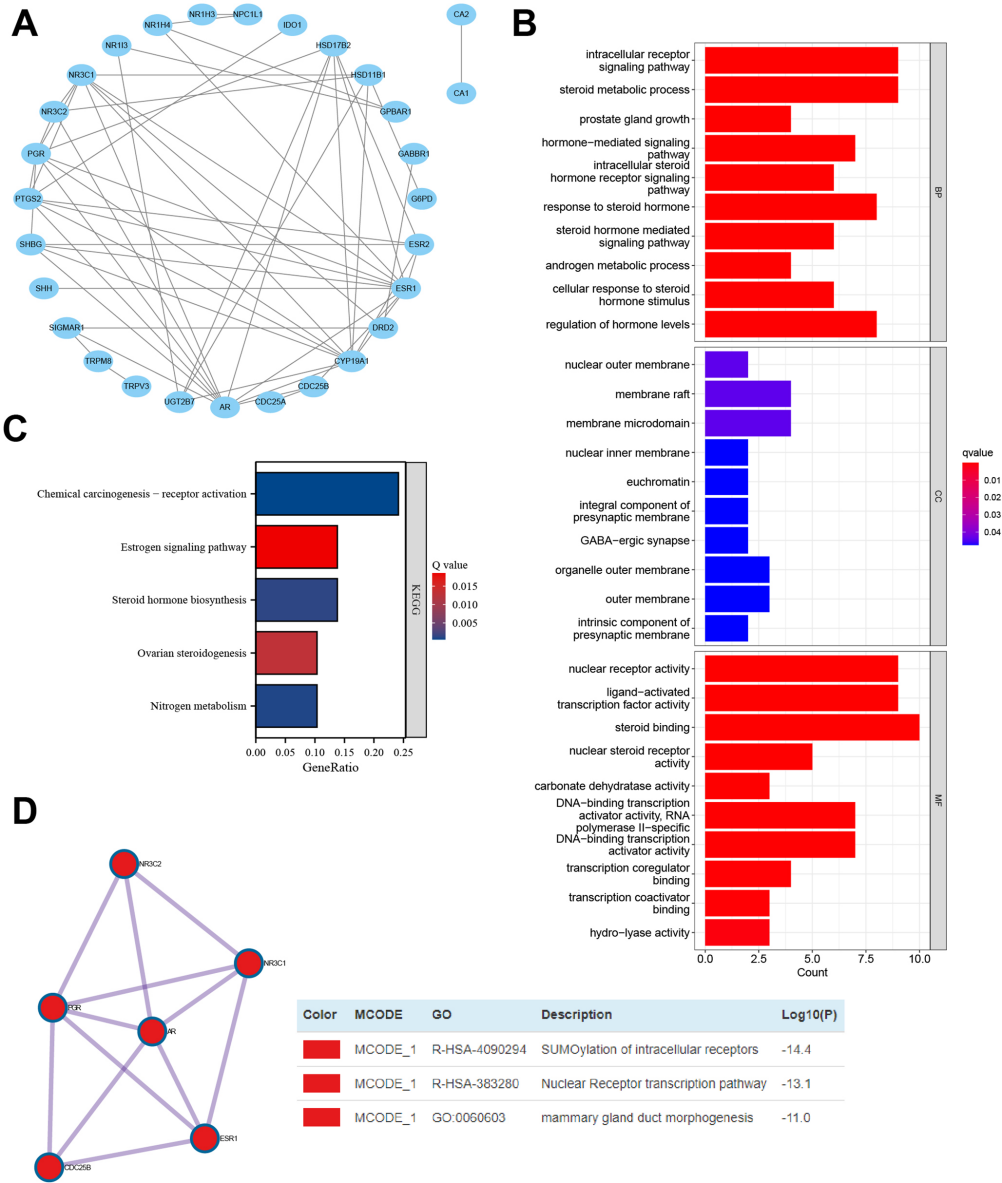
Protein-protein interaction analysis and GO/KEGG functional analysis of menthol targets Note: (**A**) Interactome network of 31 menthol target genes obtained from the SwissTargetPrediction database; (**B**) GO functional analysis results of menthol targets at the BP, CC, and MF levels; (**C**) KEGG pathway enrichment analysis results of menthol targets; (**D**) Detection results of menthol target molecular complexes (Molecular Complex Detection, MCODE) obtained from the Metascape database, with the table on the right indicating the potential BP in which these complexes may be involved

Previous studies have shown that menthol can enhance the activity of cold-exposed mice, promote thermogenesis, and reduce the severity of cold injury. To further investigate whether menthol can prevent cold injury, we sought to identify the drug targets of menthol in cold injury. Using the SwissTargetPrediction database, we predicted the target proteins of menthol and found that both isoforms, L-Menthol and DL-Menthol, shared the same targets in SwissTargetPrediction, with 31 target genes having a probability > 0. Subsequently, we imported these genes into the String database for protein interaction analysis and constructed a PPI network graph using Cytoscape software (Fig. 2A).

Furthermore, we conducted GO functional analysis and KEGG pathway analysis on the 31 target genes. The GO functional analysis results (Fig. 2B) revealed that the 31 target genes were involved in BP, such as intracellular receptor signaling pathways, steroid metabolic processes, and prostate gland growth. In terms of CC, they were mainly enriched in the nuclear outer membrane, membrane raft, and membrane microdomain. For MF, they were predominantly enriched in nuclear receptor activity, ligand-activated transcription factor activity, and steroid binding. The KEGG pathway analysis indicated that the 31 target genes are involved in signaling pathways, including Chemical carcinogenesis – receptor activation, Estrogen signaling pathway, and Steroid hormone biosynthesis (Fig. 2C).

Subsequently, we used the Metascape database to detect potential molecular complexes (Molecular Complex Detection, MCODE) within the target genes. The results showed that among the 31 target genes, there was one MCODE related to SUMOylation of intracellular receptors, the Nuclear Receptor transcription pathway, and mammary gland duct morphogenesis (Fig. 2D).

### TRPM8, as a target protein of menthol, may play a key role in preventing cold injury

**Fig. 3:**
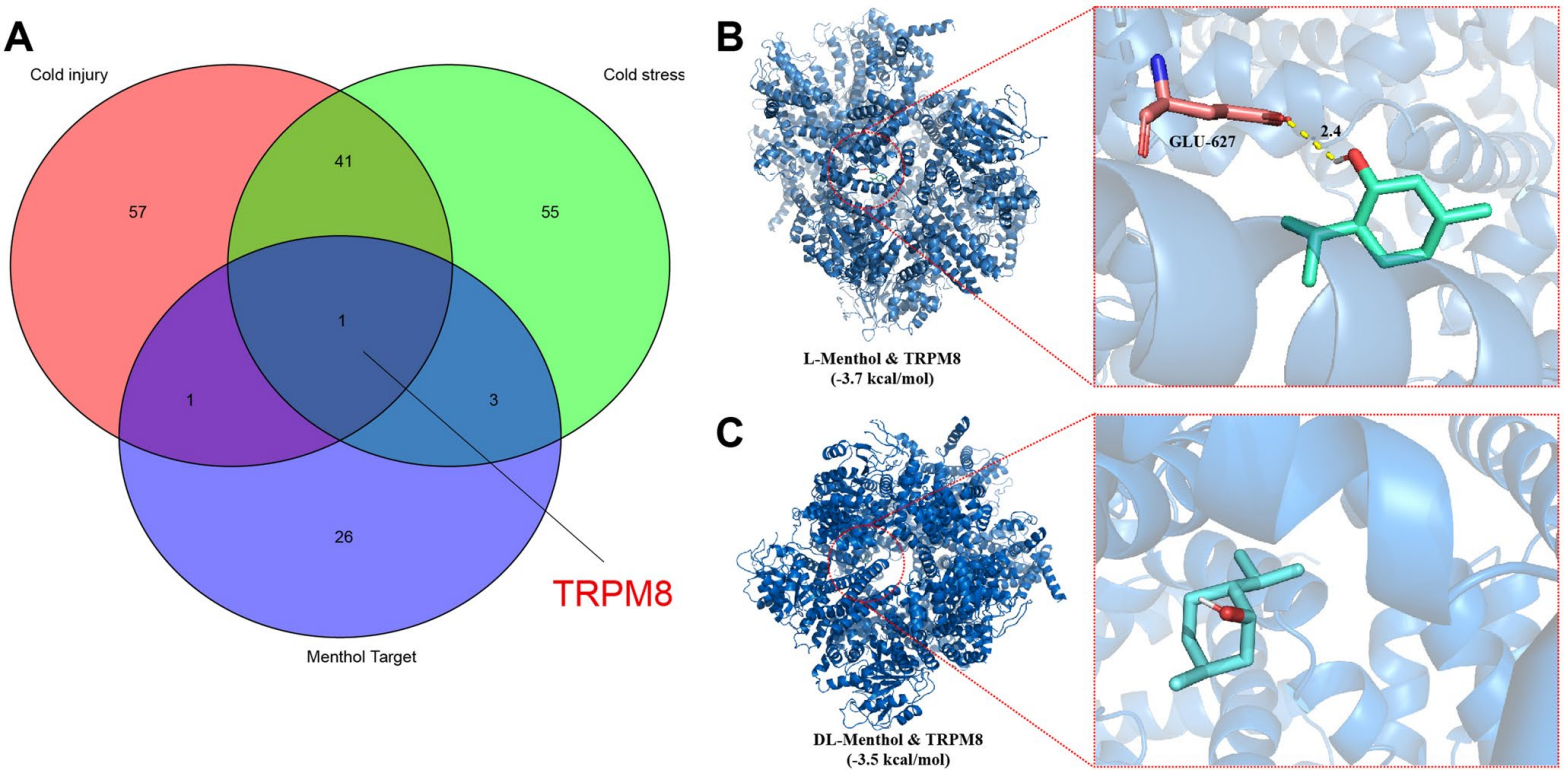
TRPM8 screening and molecular docking results with menthol Note: (**A**) Intersection between the top 100 genes related to “Cold injury” and “Cold stress” gene sets in the GenCards database and the 31 target genes of menthol; (**B-C**) Docking situations of TRPM8 with two menthol molecules, where the blue substance represents the target protein’s secondary structure, and the green molecule structure inside the red circle represents the small menthol molecule

In the quest to identify key targets of menthol for preventing cold injury, we retrieved and obtained sets of genes related to “Cold injury” and “Cold stress” from the GenCards database, yielding 3276 and 3626 respective genes. We intersected the top 100 genes based on the Relevance score from each set with 31 menthol target genes, resulting in a singular outcome: TRPM8 (Fig. 3A). Thus, we postulate TRPM8 as a critical target of menthol in preventing cold injury.

Furthermore, we conducted molecular docking analysis of TRPM8 and menthol using software such as AutoDockTools 1.5.6 and Vina 1.1.2. Binding energy < 0 kcal/mol indicates spontaneous binding and interaction of the protein molecules, with lower binding energy implying greater molecular stability [27]. Our findings reveal that both types of menthol molecules (L-Menthol and DL-Menthol) exhibit spontaneous binding with TRPM8, with binding energies of -3.7 kcal/mol and − 3.5 kcal/mol, respectively (Fig. 3B-C).

### Menthol induces BAT thermogenesis by activating the “cold channel” TRPM8

The thermogenesis mediated by UCP1 is considered to be part of the proton leak hypothesis [29]. Uncoupling oxidative phosphorylation leads to the dissipation of energy in the form of heat without producing ATP, resulting in decreased ATP levels in BAT cells [30]. Subsequently, we investigated the potential mechanism by which menthol alleviates cold injury.

**Fig. 4:**
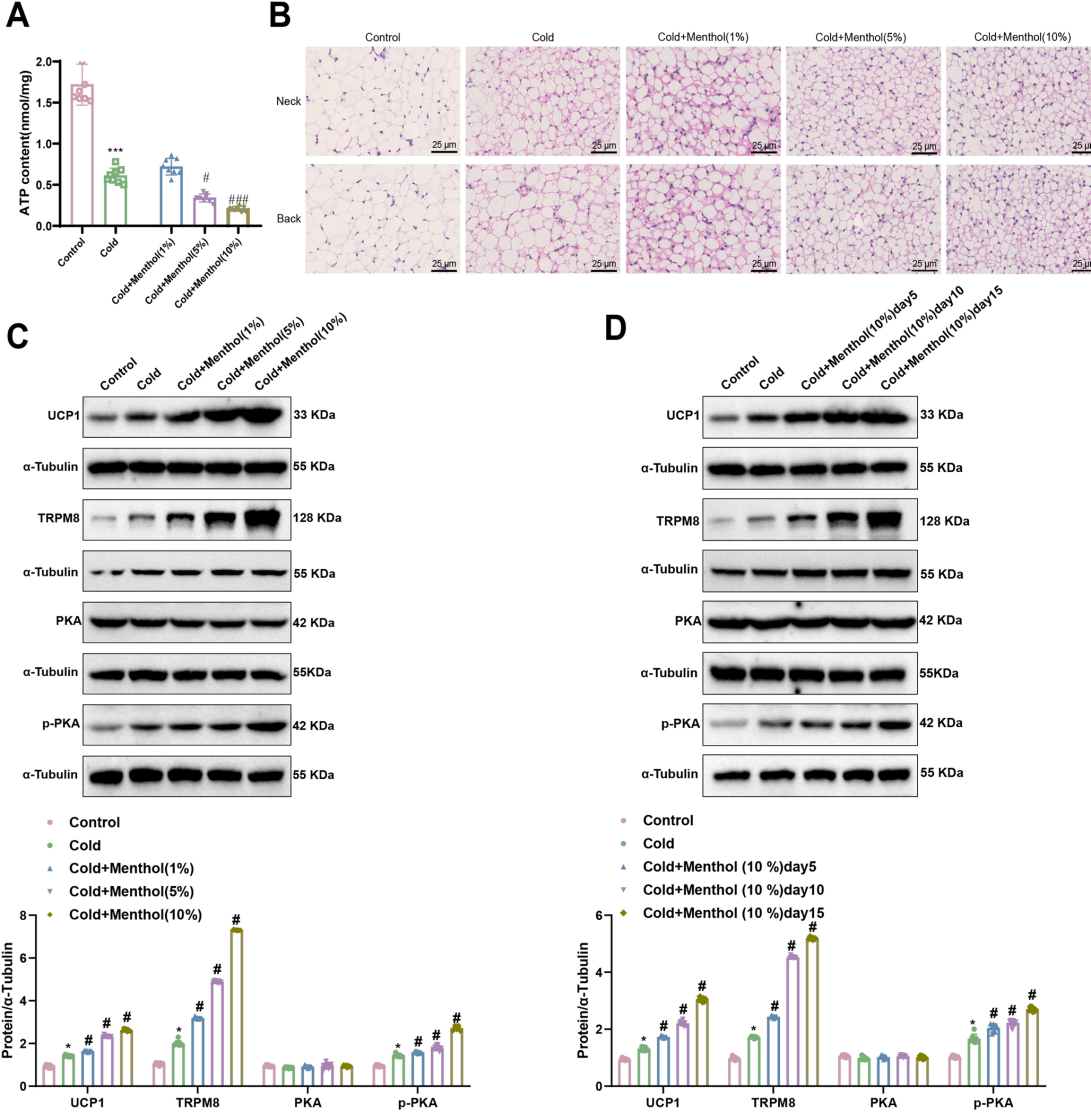
Menthol induces BAT thermogenesis by activating the “cold channel” TRPM8 Note: (**A**) ATP content in mouse BAT; (**B**) Changes in lipid droplets in mouse BAT (400×, 25 μm); (**C-D**) Western blot analysis of UCP1, TRPM8, PAK, and p-PAK protein expression in BAT, and analysis of relative band densities. Single-factor analysis of variance was used for the data, followed by Tukey’s post hoc comparison test, with mean ± standard deviation shown (*n* = 8). Compared to the Control group, *p < 0.05, ***p < 0.001; compared to the Cold group, ^#^*p* < 0.05, ^###^*p* < 0.001

As shown in Fig. 4A, the ATP levels in BAT were significantly reduced in the cold-exposed group compared to the control group, indicating that cold stimuli are potent activators of BAT thermogenesis. When comparing the low-dose (1%) menthol-treated group with the cold-exposed group, there was no statistically significant difference in ATP levels in BAT. In contrast, the medium-dose (5%) and high-dose (10%) menthol-treated groups exhibited a significant reduction in ATP levels in BAT, suggesting that menthol further promotes thermogenesis. To assess the morphological characteristics of BAT after menthol treatment, we conducted an H&E staining analysis. Additionally, as demonstrated in Fig. 4B, H&E staining of brown adipose tissue (BAT) from the neck and back regions revealed a reduction in lipid droplet volume in the cold-exposed group compared to the control group. Furthermore, the menthol treatment groups showed a gradual decrease in BAT size with increasing concentrations of menthol, indicating that menthol reduces the volume of lipid droplets and converts fat into calories. These results collectively indicate that menthol can improve the morphological structure of BAT.

Subsequently, we validated the targets through Western blot analysis. Previous results suggested that UCP1 is a key target in BAT thermogenesis, and PKA phosphorylation plays a crucial role in the thermogenic process. As illustrated in Fig. 4C, the expression of TRPM8, UCP1, and p-PKA in BAT were elevated in the cold-exposed group compared to the control group. However, treatment with medium (5%) and high (10%) doses of menthol further increased the expression of TRPM8, UCP1 and p-PKA in BAT, especially in the high-dose (10%) menthol treatment group. Given that the high-dose (10%) menthol treatment had the most significant impact on BAT thermogenesis, we opted to proceed with the high-dose (10%) menthol treatment for subsequent experiments. Additionally, the expression levels of TRPM8, UCP1, and p-PKA were further assessed on days 5, 10, and 15, with the high-dose (10%) menthol group showing continued increases in expression over time (Fig. 4D).

These findings indicate that menthol promotes BAT thermogenesis by activating TRPM8 channels in BAT cells, enhancing PKA phosphorylation.

### Menthol induces BAT thermogenesis via TRPM8 activation, enhances cold tolerance in exposed mice, and prevents cold injury

**Fig. 5:**
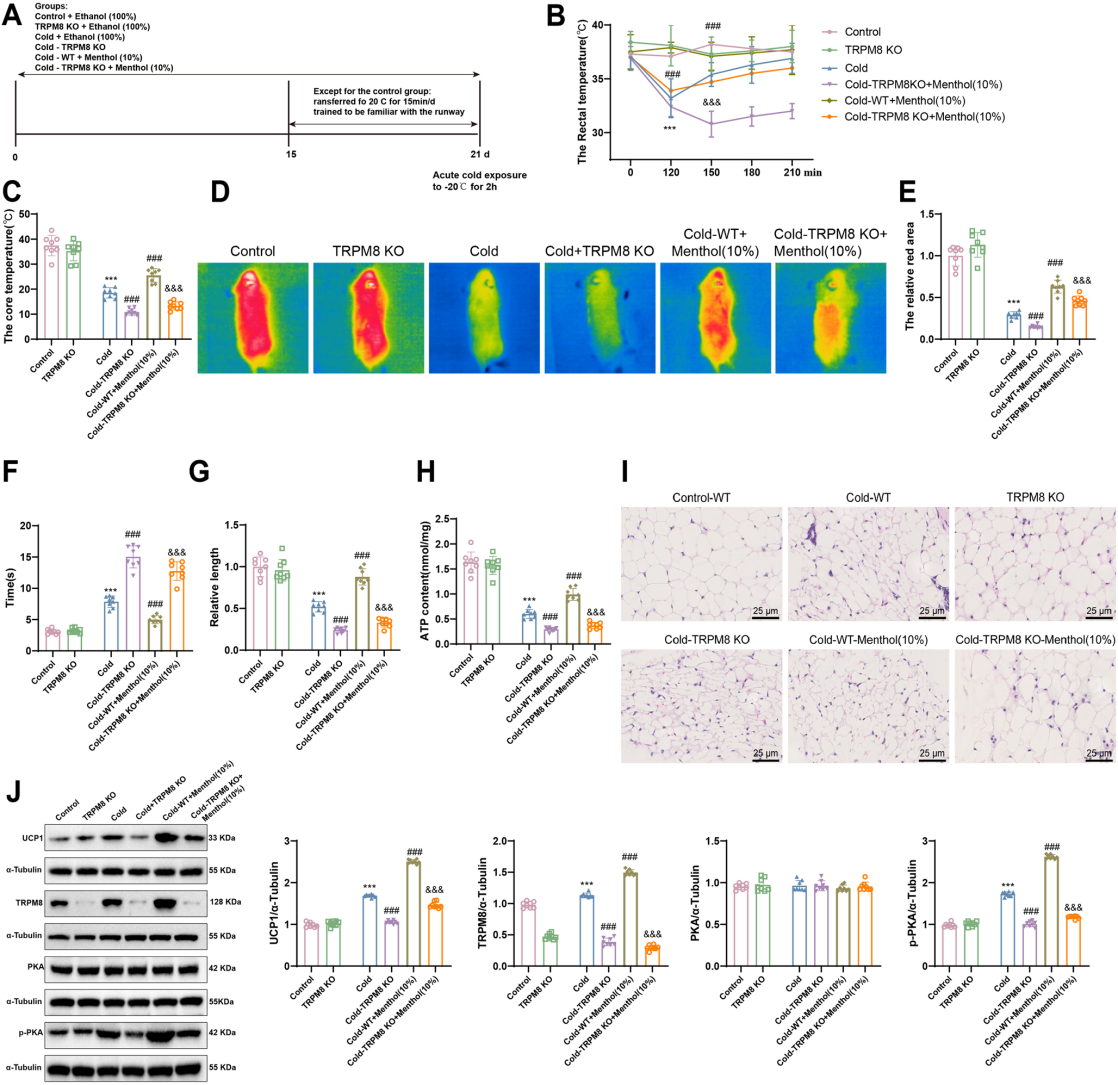
Menthol induces BAT thermogenesis by activating TRPM8, thereby enhancing cold tolerance in mice and preventing cold-induced injury Note: (**A**) Experimental design of drug administration and cold exposure. (**B-C**) Rectal temperature (**B**) and core temperature (**C**) recovery 2 h after cold exposure on day 21 of drug treatment. (**D-E**) Surface temperatures of TRPM8 knockout mice drop lower than those in the menthol-treated group after exposure to -20 °C for 2 h. Warmer colors indicate higher body temperatures, with the red area representing the highest core temperature. (**F**) Assessment of mouse activity after 120 min of cold exposure following 21 days of drug administration. (**G**) The relative tail length of each mouse group. (**H**) ATP content in mouse BAT. (**I**) Changes in lipid droplets in mouse BAT (400×, 25 μm). (**J**) Western blot bands displaying UCP1, TRPM8, PAK, and pPKA protein expression in BAT, along with the analysis of band densities. Data were analyzed using two-way ANOVA, followed by Tukey’s post hoc test, with mean ± standard deviation shown (*n* = 8). Compared to the Control-WT group, *p < 0.05, **p < 0.01, ***p < 0.001; compared to the Cold group, ^#^*p* < 0.05, ^##^*p* < 0.01, ^###^*p* < 0.001; compared to the Cold-WT-Menthol (10%) group, ^&^*p* < 0.05, ^&&^*p* < 0.01, ^&&&^*p* < 0.001

To investigate whether menthol induces BAT thermogenesis via TRPM8 activation to enhance cold tolerance in exposed mice, we established WT and TRPM8 KO mouse models. The mice were divided into the following groups: WT, WT exposed to cold, TRPM8 KO, TRPM8 KO exposed to cold, WT with high-dose menthol (10%) exposed to cold, and TRPM8 KO with high-dose menthol (10%) exposed to cold (Fig. 5A). Compared to the control group of WT mice, the rectal and core temperatures significantly decreased in the WT cold-exposed group, while no significant changes were observed in the TRPM8 KO mice. Additionally, in comparison to the cold-exposed WT group, the rectal and core temperatures significantly decreased in the TRPM8 KO mice exposed to cold, whereas the high-dose menthol-treated WT mice showed a significant increase in rectal and core temperatures following cold exposure. Furthermore, compared to the WT group exposed to high-dose menthol (10%) and cold, the TRPM8 KO mice treated with high-dose menthol (10%) maintained lower body temperatures after cold exposure (Fig. 5B-C).

Furthermore, thermal imaging showed that, in comparison to the WT control group, the surface temperatures decreased in the WT mice exposed to cold, with no change observed in the TRPM8 KO mice. When compared to the cold-exposed WT group, the TRPM8 KO mice exposed to cold exhibited lower body temperatures and could not maintain their body temperature in the cold environment, while the high-dose menthol-treated WT mice displayed improved cold tolerance after cold exposure. In contrast, when compared to the WT group treated with high-dose menthol (10%) and exposed to cold, the TRPM8 KO mice treated with high-dose menthol (10%) continued to show lower cold tolerance after cold exposure (Fig. 5D-E). Furthermore, in comparison to the WT control group, the cold-exposed WT mice spent more time crossing the runway, indicating a gradual decrease in physical activity, whereas there was no significant change in the time spent by the TRPM8 KO mice crossing the runway. Moreover, compared to the cold-exposed WT mice, the TRPM8 KO mice exposed to cold took longer to cross the runway, suggesting a gradual decline in physical activity, while the WT mice treated with menthol showed a reduced time crossing the runway after cold exposure, indicating an improvement in physical activity. Finally, when compared to the WT group treated with high-dose menthol (10%) and exposed to cold, the TRPM8 KO mice treated with high-dose menthol (10%) displayed weaker physical activity after cold exposure (Fig. 5F). Relative tail length was shorter in the cold-exposed WT mice compared to the WT control group, with no significant change observed in the relative tail length of the TRPM8 KO mice. However, treatment with menthol in the WT mice resulted in a longer relative tail length after cold exposure in comparison to the cold-exposed WT group. Conversely, compared to the WT group treated with high-dose menthol (10%) and exposed to cold, the TRPM8 KO mice treated with high-dose menthol (10%) had a shorter relative tail length after cold exposure (Fig. 5G). Furthermore, the TRPM8 KO mice still exhibited a shorter relative tail length after cold exposure compared to the WT cold-exposed group. Overall, these results demonstrate that menthol, through TRPM8 activation, induces BAT thermogenesis to enhance cold tolerance in exposed mice and prevent cold injury.

To investigate the mechanism by which menthol activates TRPM8 to induce BAT thermogenesis, we assessed the ATP content of BAT (Fig. 5H). The results showed that compared to the control group of WT mice, the BAT ATP content decreased in WT mice exposed to cold, with no significant change observed in TRPM8 KO mice. Furthermore, post-cold exposure, the ATP content in BAT significantly decreased in TRPM8 KO mice compared to cold-exposed WT mice. Conversely, WT mice treated with a high dose of menthol (10%) showed a significant increase in BAT ATP content post-cold exposure. Additionally, when compared to the WT-high dose menthol (10%)-cold exposure group, the TRPM8 KO mice treated with a high dose of menthol (10%) exhibited a decrease in BAT ATP content post-cold exposure.

H&E staining results revealed that compared to the control WT mice group, the BAT volume in cold-exposed WT mice decreased, while no significant change was observed in TRPM8 KO mice. Moreover, both TRPM8 KO mice and WT mice treated with a high dose of menthol (10%) continued to exhibit a decrease in BAT volume post-cold exposure compared to cold-exposed WT mice. Interestingly, when compared to the WT-high dose menthol (10%)-cold exposure group, the TRPM8 KO mice treated with a high dose of menthol (10%) showed a slight increase in BAT volume (Fig. 5I).

Additionally, compared to the control group of WT mice, the expression levels of TRPM8, UCP1, and p-PKA proteins in BAT of WT mice exposed to cold were elevated, while in TRPM8 KO mice, the expression of TRPM8 was reduced, and no significant changes were observed in the expression of UCP1, and p-PKA proteins. Compared to the cold-exposed WT mice, the protein expression of UCP1, and p-PKA decreased in TRPM8 KO mice after cold exposure, but in WT mice with TRPM8 knockout and in WT mice treated with a high dose of menthol (10%), the protein expression of TRPM8, UCP1, and p-PKA all increased further after cold exposure. Moreover, compared to the WT group exposed to high-dose menthol (10%) and cold, the expression levels of these proteins decreased in TRPM8 KO mice treated with high-dose menthol (10%) after cold exposure (Fig. 5J).

These findings suggest that menthol activates the TRPM8 channel in brown adipocytes, promotes PKA oxidation and phosphorylation, and induces BAT thermogenesis.

## Discussion

Menthol, as a partial agonist of the cold-activated TRPM8 receptor [8], can promote cold exposure in mice by binding to the TRPM8 receptor, inducing a stress response simulating environmental cold exposure in the mice [9, 31]. This process can alleviate the pain caused by cold injury [32], reduce levels of inflammatory and chemotactic factors to alleviate inflammation infiltration, exhibiting anti-inflammatory effects [19, 33]. Additionally, it increases the activity of antioxidant enzymes SOD, GR, and GPx, as well as the levels of GSH, ameliorating oxidative stress-induced damage from reperfusion [10]. Furthermore, activation of the TRPM8 channel in BAT cells induces direct thermogenesis by UCP1 in mitochondria, enhancing resistance to cold temperatures [34]. Our study also confirmed that knocking out TRPM8 decreased the cold tolerance in mice, while menthol enhanced the cold tolerance in mice exposed to cold temperatures.

Based on network pharmacology and bioinformatics analysis, our research found that menthol can promote brown fat thermogenesis by activating TRPM8. These results indicate that menthol binding to the TRPM8 receptor triggers a cold sensation in the body, prompting a reflexive response to cold stimuli. This activation regulates PGC-1α through the PKA pathway in BAT, activating thermogenesis genes, and UCP1 transfers protons to the mitochondrial matrix for thermogenesis to counter external cold stimuli. The TRPM8 channel can be activated by temperature changes [35], leading to increased UCP1-dependent BAT thermogenesis [36]. Studies also suggest that under certain stimuli, brown fat produces 12-LOX to enhance 12-HEPE activation of the PI3K/mTOR/Akt/Glut pathway, providing fatty acid thermogenesis for UCP1 activation [12]. Thyroid hormones stimulate UCP1 expression and thermogenesis directly through an autophagy-dependent mechanism in peripheral BAT [37]. The cGMP/AKT pathway can influence BAT thermogenesis through NO and natriuretic peptides [38]. Recombinant human bone morphogenetic protein-4 (BMP4) can promote differentiation of human adipose stem cells into brown adipose cells [39], with the BMP family also promoting thermogenesis in brown adipose cells [40]. Menthol can induce the browning of white fat cells and enhance the activation of brown adipose cells [41]. Furthermore, the low-temperature animal model we used holds significant physiological relevance: (1) Physiological significance of simulating acute cold stress: Short-term exposure to extreme cold can simulate acute cold stress, a common environmental challenge. Studying acute stress responses helps us understand how the body activates protective mechanisms in a short period to maintain body temperature and energy balance [42]. (2) Studying the physiological mechanisms of rapid responses: In the short term, the body primarily responds to cold through shivering thermogenesis and non-shivering thermogenesis (NST) [43]. NST is mainly mediated by BAT, and studying this process can reveal the rapid activation mechanisms of BAT. Additionally, acute cold exposure rapidly activates the sympathetic nervous system, releasing norepinephrine to stimulate BAT thermogenesis. This rapid response mechanism is an important model for studying energy metabolism and thermoregulation [44].

In conclusion, our data indicate that menthol treatment can maintain mouse temperature by enhancing BAT thermogenesis, promoting rapid elevation of body temperature to escape from the cold state. Menthol exhibits a thermogenic effect, likely attributed to the activation of TRPM8 channels in BAT cells, promoting BAT thermogenesis and preventing cold injury. This study provides theoretical and experimental evidence for how menthol affects organism thermogenesis through TRPM8 receptors in BAT and how non-thermogenic pathways prevent cold injury, offering research directions and insights for the development of drugs for cold injury treatment and supporting future pharmaceutical applications.

A limitation of this study is that menthol is only a partial agonist of TRPM8 receptors, and TRPM8 receptor activation can occur through various means such as low temperatures, menthol, phosphatidylinositol (PI), and phospholipase C (PLC). In brown adipose cells, PKA-induced phosphorylation induces mitochondrial UCP1 thermogenesis; however, this pathway is not the sole thermogenesis pathway in brown adipose cells, as there are diverse pathways for thermogenesis. Additionally, TRPM8 receptors are present in white adipose cells, suggesting that menthol can activate TRPM8 receptors in white adipose cells to induce thermogenesis. With multiple confounding factors, relevant experiments should be designed, including comparing thermogenesis proportions in white and brown adipose cells in response to menthol. Future research should identify specific compounds generating thermogenic effects to elucidate their mechanisms of action and investigate target proteins and signaling pathways.

## Supplementary Information

Below is the link to the electronic supplementary material.

Supplementary Material 1

## Data Availability

All data can be provided as needed.
