# Supplementary material for: TRPM8-driven thermogenesis by menthol: mechanisms of cold injury prevention
Source: J Physiol Biochem. 2025 Sep 13;81(4):1051–66. doi: 10.1007/s13105-025-01120-8 (PMC12738656; doi:10.1007/s13105-025-01120-8)
Supplement: Supplementary file 1 — Supplementary Material 1 [file 13105_2025_1120_MOESM1_ESM.docx]

**Table S1. Western Blot Antibody Information**

| **Protein Name** | **Item No.** | **Dilution ratio** | **Brands** | **Molecular weight size** |
| --- | --- | --- | --- | --- |
| anti-TRPM8 | PA1-46239 | 1:1,000 | Invitrogen | 128 kDa |
| anti-UCP1 | ab234430 | 1:1000 | abcam | 33 kDa |
| anti-PKA | #4782 | 1:1000 | Cell Signaling | 42 kDa |
| anti-p-PKA | #4781 | 1:1000 | Cell Signaling | 42 kDa |
| α-Tubulin | ab7291 | 1:5000 | abcam | 50 kDa |
